# Wilson’s Disease with Acute Hepatic Onset: How to Diagnose and Treat It

**DOI:** 10.3390/children11010068

**Published:** 2024-01-06

**Authors:** Valeria Delle Cave, Fabiola Di Dato, Raffaele Iorio

**Affiliations:** Department of Translational Medical Science, Section of Pediatrics, University of Naples Federico II, 80131 Naples, Italy; valeria.dellecave@unina.it (V.D.C.); fabiola.didato@unina.it (F.D.D.)

**Keywords:** acute hepatitis, acute liver failure, liver transplantation, copper, ceruloplasmin

## Abstract

Wilson’s disease (WD) with acute onset poses a diagnostic challenge because it is clinically indistinguishable from other acute liver diseases. In addition, serum ceruloplasmin and urinary copper excretion, the first-line diagnostic tools for WD, can show false positive results in the case of acute liver failure, and the diagnostic role of genetic analysis is limited by the time required to perform it. In the case of fulminant onset, there is a clear indication of liver transplantation. “New Wilson Index” is frequently used to discriminate between patients who need liver transplantation versus those who can be successfully managed by medical treatment, but its reliability remains controversial. Timely referral of patients with acute liver failure due to WD may be a key factor in improving patient survival. Although liver transplant very often represents the only chance for such patients, maximum effort should be made to promote survival with a native liver. The management of these aspects of WD is still a matter of debate and will be the subject of this review.

## 1. Introduction

Wilson’s disease (WD) is a rare autosomal recessive disorder due to mutations in the ATP7B gene [1,2]. The ATP7B protein is involved in biliary copper excretion and its deficit or reduced function [3,4] results in the progressive accumulation of copper in the liver and other organs [5,6]. WD is estimated to affect approximately 1:30.000 individuals [7,8,9] and occurs in 6–12% of patients younger than 40 years hospitalized with acute liver failure (ALF) [10].

Initially described as hepatolenticular degeneration, WD clinical onset can be various, mainly affecting the liver, nervous system, and eye [5,11]. Other manifestations include renal, cardiac, and musculoskeletal diseases. The reasons why some patients present with liver disease and others with neurological or psychiatric manifestations are not well known [12]. In childhood, the manifestations are usually predominantly hepatic: they range from an incidental increase in liver enzymes without symptoms [13] to an ultrasound finding of a brilliant liver to acute and chronic clinical pictures of liver disease up to cirrhosis and its complications [11,14]. Acute liver disease due to WD is clinically indistinguishable from that due to other etiologies and may rarely be fulminant [15,16,17]. If WD is not recognized and adequately treated, the progression of hepatic and neurologic damage can be very rapid [18]. If WD is diagnosed and treated early in childhood, it is possible to avoid neurological involvement and ensure the patient a survival similar to that of the general population with a good quality of life [19]. Unfortunately, diagnosis remains a challenging patchwork involving clinical, laboratory, histological, and molecular tools [5]. When WD presents with acute liver disease or ALF, a careful differential diagnosis with all conditions (infectious, autoimmune, metabolic, toxic, and biliary disorders) that can lead to acute liver disease must be made [20]. In this case, especially in fulminant cases, the diagnostic pitfalls are multiple in contrast with the need to quickly reach a correct diagnosis. Finally, when approaching the patient with suspected WD, some inherited disorders potentially impairing hepatic copper metabolism, such as congenital disorders of glycosylation and MEDNIK syndrome, must be considered [21,22] although these diseases are not usually associated with an acute onset. The aim of this narrative review is to focus on the available evidence about the clinical presentation, diagnostic criteria, and therapeutic management of children with acute hepatic onset of WD. 

## 2. Search Strategy and Selection Criteria

In this narrative review, a systematic literature search was conducted in PubMed, Cochrane, and Google Scholar for all the literature published up to September 2023. The used keywords were “Wilson’s disease” and “Wilson disease” combined with additional terms including “acute liver disease”, “acute liver failure”, “cirrhosis”, “chelation”, “penicillamine”, “trientine”, “zinc”, “plasmapheresis”, and “liver transplantation” to identify relevant studies. Reference lists from identified articles were also assessed for relevance. Articles in languages other than English, animal studies, and abstracts presented only in conference proceedings were excluded.

## 3. Acute Clinical Presentation of WD

WD should be excluded in all children older than 1 year with signs of liver disease [4]. WD presentation with acute liver disease is characterized by symptoms such as jaundice, hypocolic stools, hyperchromic urine, abdominal pain, vomiting, anorexia, and asthenia [23]. None of them are pathognomonic of WD. In some cases, ALF may be the presenting manifestation [16]. According to the Pediatric Acute Liver Failure Study Group criteria [24], it is defined in the presence of the following criteria: i. acute onset of liver disease without evidence of chronic liver disease; ii. biochemical evidence of severe liver injury; iii. coagulopathy not responsive to vitamin K administration. The latter criterion is defined by the international normalized ratio (INR) ≥ 2.0 regardless of hepatic encephalopathy or INR ≥ 1.5 if encephalopathy is present. Although a prerequisite for the definition of ALF is the absence of previous severe fibrotic or cirrhotic chronic liver disease, WD is an exception category because, in patients with fulminant WD, underlying cirrhosis can be found [25,26].

In all children with acute liver disease, extensive etiologic research is mandatory. The potential etiology is different depending on the age of the patients (for example, genetic–metabolic causes other than WD prevail in the first years of life [27], while paracetamol intoxication and WD are more common in adolescents) [20,28]. Certainly, it is desirable to always exclude infectious causes, drug-induced liver injury, biliary, vascular, and ischemic disorders, infiltrative causes, and autoimmune liver diseases [20]. For the latter, it must be taken into account that positive autoantibodies can also be found in WD and viral infections [29,30].

According to the Pediatric Acute Liver Failure registry, WD represents approximately 3% of pediatric ALF cases [25]. This condition is the most dramatic and life-threatening WD presentation and occurs more frequently in children and young adults than later ages [31], with a female/male ratio of 4:1 [32,33]. This is likely due to an increased susceptibility related to sex hormones, as demonstrated by studies conducted on an animal model of WD [34]. The accumulation of copper above a toxic threshold induces hepatocellular necrosis with subsequent release of the metal into circulation and an increase in serum-free copper levels, resulting in renal failure, neurological symptoms, and hemolysis [35], features usually defined as Wilson’s crisis [36]. Fulminant Wilson’s disease carries 95% mortality if left untreated [37,38], and it is nearly always fatal without liver transplantation [39]. Coombs-negative hemolytic anemia can be the first manifestation of ALF due to WD (ALF-WD), characterized by the presence of severe non-immune intravascular hemolysis [40,41], sometimes apparently precipitated by infections or drugs. Therefore, a practical guide from the British Association for the Study of the Liver recommended that all patients with unexplained Coombs-negative hemolytic anemia and/or movement disorders [42] should be investigated for WD [43]. ALF-WD accompanied by hemolytic crisis is estimated to occur in 30% of children with ALF who require a liver transplant and in 60% of those with unfavorable evolution before transplantation [44].

In summary, in the case of acute liver disease, warning signs to suspect ALF-WD are rapidly developing jaundice, Coombs-negative hemolytic crisis, coagulopathy unresponsive to vitamin K administration, rapid progression of renal failure, and neurological deterioration. If liver transplantation is not performed, there is a high mortality risk [45]. A rapid worsening of liver function may also occur in WD patients who have previously been treated but who have decided to discontinue drug treatment [46,47]. WD can present as ALF also in patients with pre-existing unrecognized chronic liver disease [20]. In some of these cases, viral infections can be a precipitating event [27]. The genotype–phenotype correlation in WD is still a matter of debate and does not explain the acute manifestations of the disease [12].

## 4. Diagnostic Challenges

A reliable diagnosis of WD requires a combination of clinical, laboratory, histological, and molecular tests [5], so in 2003, Ferenci et al. [48] proposed a diagnostic score for WD, which is still used [49] (Table 1).

### 4.1. Serum Ceruloplasmin

The first step for WD diagnosis is the assessment of ceruloplasmin whose serum level is reduced for impaired biosynthesis. Up to 20% of pediatric and adult WD patients have normal ceruloplasmin levels [14,50]. On the other side, hypoceruloplasminemia is not always indicative of a copper storage disorder. Indeed, both heterozygotes for WD and patients with other disorders such as decompensated liver failure and congenital disorders of glycosylation [51] may share this feature [52]. Furthermore, it is also shared by the genetic condition aceruloplasminemia, characterized by the abnormal accumulation of iron in the brain and various organs. Although there is still debate about the best diagnostic threshold for serum ceruloplasmin, 20 mg/dL is still universally accepted [53]. However, in patients with acute hepatic failure, a serum ceruloplasmin cut-off value of 16 mg/dL resulted in the highest diagnostic accuracy [54]. Enzymatic assay to evaluate ceruloplasmin level is to be preferred over the immunoassays that are based on indirect methods and may overestimate the amount of ceruloplasmin [55].

In ALF-WD or patients with acute hepatitis, the serum concentration of ceruloplasmin is less reliable [56] because it may be falsely elevated or normal under the influence of inflammation [57,58]. Furthermore, serum ceruloplasmin levels may also be reduced in children with ALF for causes other than WD as a result of impaired hepatic proteosynthetic function.

### 4.2. Urinary Copper Excretion

Urinary copper excretion represents, with ceruloplasmin, the first level test for WD diagnosis. The urinary copper level seems to be directly related to age at WD diagnosis: in children, the accepted threshold for diagnosis is 40 μg/24 h [18], while in adults, it is 100 μg/24 h [5].

In the ALF setting, urinary copper excretion poses interpretation issues such as ceruloplasmin. In fact, urinary copper excretion above the normal range is common in non-WD ALF due to the massive necrosis of hepatocytes and the subsequent passive release of copper from hepatocytes. On the other side, although patients with ALF-WD may have a basal urinary copper content up to 30 times the upper limit of normal [16,59,60], a specific threshold for ALF-WD has never been defined and moderately positive values cannot be used as a sole tool for WD diagnosis. Urinary copper excretion measurement after penicillamine challenge has been less commonly used in recent years [34]. It is performed by collecting 24 h urine samples giving five hundred milligrams of D-penicillamine immediately before and after 12 h of the collection. In the initial study by Martins da Costa et al. [61], it was performed in children and the diagnostic threshold was set at 1600 μg/24 h (25 μmol/24 h). When Ferenci et al. [48] established the test positivity threshold as part of their diagnostic score, they indicated values > 5 × the upper limit of normal (ULN) as positive. Although not explicitly stated, since the normal upper limit is 100 μg/24 h (2 μmol/24 h) for adults and 40 μg/24 h (6.04 μmol/24 h) for children, these thresholds correspond to 500 μg/24 h (8 μmol/24 h) and 200 μg/24 h (3.2 μmol/24 h), respectively. However, for this test there is no strong evidence to support its use in an ALF setting; furthermore, it appears inadvisable to avoid too rapid mobilization of copper by a high chelating dose.

### 4.3. Total Serum Copper and Non-Ceruloplasmin Bound Copper (NCC)

Total serum copper concentration measures two different pools of copper in the blood: that associated with ceruloplasmin, not toxic, and the “free copper”, which represents the toxic fraction of the metal [62,63,64]. In asymptomatic WD children, serum copper is usually reduced due to low levels of ceruloplasmin. On the one hand, a reduced copper concentration may be also found in heterozygous WD carriers [65,66]. On the other hand, the diagnostic role of serum copper in ALF-WD is also compromised by the possible occurrence of elevated levels following the sudden and massive release of copper due to hepatic necrosis [16,67,68]. Moreover, free copper, then called non-ceruloplasmin bound copper (NCC), may be elevated in acute hepatic failure of any etiology, in chronic cholestasis, or in copper intoxication [69]. In most untreated patients or non-adherent, it is elevated above 25 µg/dL, while NCC concentration < 5 µg/dL may indicate systemic copper depletion [4]. NCC has been proposed as a diagnostic tool but also as a monitoring parameter in WD patients. Some mathematical formulas have been proposed to calculate the fraction of free copper, but they are unreliable and inaccurate due to immunological and enzyme-based assays of ceruloplasmin [4,11,54,55]. For this reason, a direct measurement of the NCC is desirable. Firstly, the exchangeable copper test (CuEXC) and the relative exchange copper (REC), reflecting the ratio of CuEXC to total serum copper, have been shown to be an accurate tool for the diagnosis of WD, but [70,71] very few laboratories currently perform this test [72] and there is no experience on a large series [64]. Nowadays, several techniques are being developed for a direct evaluation of the toxic fraction of the metal that seems to be an early predictor of clinical treatment failure. Based on the hypothesis that free copper is in equilibrium with tissue copper, NCC in the near future is expected to become the best marker for monitoring treatment, predicting failure or poor adherence in advance, and avoiding overtreatment [55].

### 4.4. Other Clues to the Diagnosis of ALF-WD

Laboratory tests in ALF-WD usually show hyperbilirubinemia with values that can exceed 300 µmol/L (17.5 mg/dL), relatively low serum transaminases levels (100–500 IU/L) [35,73], low alkaline phosphatase (usually <40 IU/L) [74,75], low uric acid, and reduced liver synthesis indices [11]. There is usually a disproportion between the low serum alkaline phosphatase levels and the high total bilirubin levels resulting from the concomitant increase in indirect bilirubin due to copper-induced hemolysis. While in hepatitis due to infections, autoimmune hepatitis, or drug-induced hepatotoxicity, ALT is often more elevated than AST, in ALF due to WD, AST levels are usually higher than ALT, likely due to mitochondria function impairment [76]. The relatively modest increase in serum aminotransferase activity seen in most ALF-WD patients, compared with ALF due to other etiologies, often leads to an underestimation of disease severity [11]. The blood count may show thrombocytopenia due to hypersplenism and anemia that could also be secondary to Coombs-negative hemolytic anemia [77].

Korman et al. [16] used alkaline phosphatase/total bilirubin ratio (AP/TB) < 4 and aspartate transaminase/alanine transaminase ratio (AST/ALT) > 2.2 in early identification of ALF due to WD. They reported excellent sensitivity and specificity confirming that serum ceruloplasmin and serum copper levels are less reliable in identifying patients with ALF-WD. In children where AP levels are higher because of the bone component, AP/TB accuracy is limited [78]. A positive AST/ALT and AP/TB ratio strongly suggest ALF-WD, but a negative result does not exclude the diagnosis.

**Figure 1:**
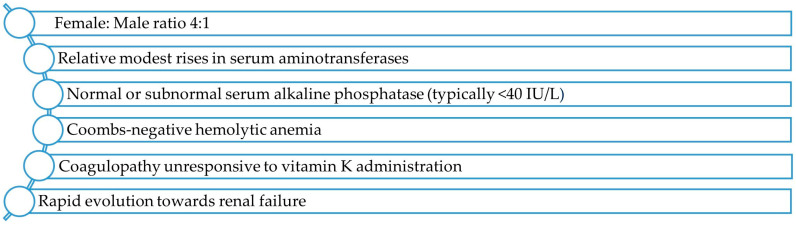
Characteristic pattern of ALF-WD. Data from Refs [14.^32^].

In brief, most patients with ALF-WD present a characteristic pattern [14,32] (Figure 1).

In the setting of ALF-WD, Kayser–Fleischer (KF) rings are detectable in up to 50% of patients. Although the combination of KF rings and low ceruloplasmin is considered the best indicator for a rapid diagnosis, KF rings are rare in the pediatric population [14,79].

Some authors evaluated the possible use of serum zinc levels as a new surrogate marker not only to facilitate WD diagnosis but also to assess disease severity with sensitivity and specificity values of 87% and 99%, respectively [80]. However, nowadays, this test is not routinely used in the common clinical practice of patients with suspected ALF-WD.

Genetic analysis of *ATP7B* has a crucial role in WD diagnosis [81] because the finding of two causative mutations confers a score of 4 on the Ferenci score [48], which is equivalent to a definitive WD diagnosis. Unfortunately, although it may be the optimal tool for the early diagnosis of ALF-WD, its usefulness is limited by the time it takes to perform.

### 4.5. Wilson’s Disease Versus Autoimmune Hepatitis Diagnosis

The diagnosis of WD can also be complicated by the detection of positive autoantibodies characteristic of autoimmune hepatitis (AIH). In fact, a low titer of autoantibodies such as anti-nuclear antibodies (ANA) and anti-smooth muscle antibodies (SMA) has been found in patients with WD without AIH [82,83], but their prevalence and significance are not known [84,85]. The presence of autoantibodies, sometimes, can lead to a false diagnosis of autoimmune disease. Some authors suggested that the appearance of these autoantibodies in WD may be induced by hepatocyte necrosis, especially in the early stages of the disease [86]. A recent study by Jańczyk W et al. [87] found that most WD patients (84%) had positive titers for ANA, SMA, anti-parietal cell antibodies (APCA), anti-neutrophil cytoplasmic antibodies (ANCA), or a combination of autoantibodies, with a highly positive reaction (title 1:160 and above) in 37% of cases. In contrast, only 28% of healthy controls presented a positive autoantibody titer. In brief, WD may be difficult to distinguish from AIH in some circumstances, particularly in the pediatric population [84,88,89] and in the context of ALF presentation. Thorough screening for WD is therefore recommended in patients with an initial diagnosis of AIH.

### 4.6. Liver Biopsy in ALF-WD

For diagnostic purposes, liver biopsy in children is only required if a definite diagnosis of WD is not achieved with non-invasive tests or if further liver disorders are suspected. In cases where there is coagulopathy, however, the use of liver biopsy is usually precluded. Liver biopsy of WD patients may show a wide range of features such as moderate to severe steatosis, varying degrees of portal and/or lobular inflammation, and fibrosis up to cirrhosis. Further findings include liver cell degeneration and ballooning, Mallory hyaline bodies, liver cell necrosis [90], and glycogenation of periportal hepatocytic nuclei. None of these lesions are specific for WD [91]. Hepatic copper accumulation is the hallmark of WD, and liver copper content greater than 250 μg/g dry weight is considered diagnostic for WD. However, there are other cholestatic disorders in which hepatic copper content may be increased above this level. Values < 50 μg/g dry weight exclude the diagnosis of WD [18].

Some conditions such as congenital glycosylation disorders, MEDNIK syndrome, manganese transport defects, and multidrug resistance protein 3 (MDR3) deficiency [21] may strongly resemble WD in terms of low serum copper and/or ceruloplasmin levels, copper accumulation on liver biopsy, and increased copper excretion [22,47,92], but these conditions are not usually associated with an acute onset of disease. For this reason, despite all the aforementioned clues, the diagnosis of WD still remains a tortuous challenge, even more so in the setting of ALF.

## 5. Prognostic Score Systems

Prognostic scoring systems have been created separately for ALF in the context of WD or non-WD to help differentiate between who would have a poor survival outcome without a liver transplant by who could have a good outcome with only medical therapy [93].

In 1986, Nazer et al. [94] proposed an ALF-WD scoring system to predict the outcome in both adult and child patients. This score included the following findings: serum bilirubin, AST, and prothrombin time (INR) (Table 2). A score ≥ 7 was predictive of death, whereas a score ≤ 6 was predictive of survival with chelation therapy [36].

In 2005, Dhawan et al. proposed a new scoring system, known as the “New Wilson Index” (NWI), analyzing a larger sample of exclusively pediatric patients and utilizing the multi- and univariate analysis of risk factors predictive of liver disease severity [14]. This score, like that of Nazer, assigns from 1 to 4 points for each variable, while it includes other parameters such as white blood cell count and serum albumin (Table 2). Within the score, serum levels of bilirubin and AST are markers of liver disease, while serum albumin reflects the ability of hepatic proteosynthesis and INR values are indicators of acute liver failure. White blood cell count is related to systemic inflammatory response [95]. The NWI should be applied for the prognostic evaluation and decision of liver transplantation in ALF-WD children: when ≥11, it is highly probable that the patient will need a liver transplant; when <11, the patient has a better chance of surviving with the native liver. Patients with an NWI of 8–10 require careful monitoring for at least 2 months because liver transplantation may be necessary if WD worsens despite pharmacological therapy [96].

There is no agreement on the accuracy of the different available score systems [97]. The NWI score was prospectively evaluated by Chanpong A et al. [96] over a period of 13 years in 52 children with WD with a median age at diagnosis of 11.69 years (range 3.92–17.26 years), still representing a good predictor for liver transplantation in patients with ALF-WD, providing a sensitivity of 80%, a specificity of 100%, and a positive-predictive and negative-predictive value of 100% and 80%, respectively. The NWI was also validated in adults [98] and showed good positive predictive value for mortality without liver transplantation with a sensitivity of 93%, a specificity of 98%, and a positive predictive value of 93% [14]. On the other hand, a review by Proost et al. reported that 46% of 37 patients (aged 5–30 years) with ALF-WD who had an NWI ≥ 11 survived with plasmapheresis without liver transplantation [99]. Moreover, the authors focused their attention on 17 patients with an NWI ≥ 11 described in the literature, successfully treated with plasmapheresis without liver transplantation, showing a low specificity (15%) of the score [99]. 

## 6. Treatment

The overall therapeutic goal of WD is to block or reverse organ damage related to copper accumulation. This can be achieved through medical therapy or, in case of non-response, through liver transplantation. For WD patients, lifelong medical therapy is required. It includes treatment with copper chelators (penicillamine or trientine) or zinc salts according to available guidelines [4,23,40] (Table 3). Chelators mobilize intracellular copper and increase its urinary excretion. Moreover, D-penicillamine may also induce the release of intracellular stores of metallothionein, an endogenous copper chelator [100], while trientine seems able to inhibit dietary copper absorption [55]. Zinc salts induce copper-binding metallothioneins in enterocytes, lowering copper intestinal absorption into the portal circulation, and in hepatocytes, decreasing the toxic effects of free copper by trapping it within the cells [101,102]. Treatment for WD patients depends on the clinical phenotype and should be individualized, based on the severity of symptoms [103].

In WD patients, the development of fulminant liver failure appears to be related to the excess of serum copper that damages the hepatocytes, the red blood cell membrane with subsequent intravascular hemolysis, and the damage of tubular cells until the development of renal failure. Various methods have been used to reduce the acute copper load with variable clinical results. Although it is reported that ALF-WD patients with an NWI ≥ 11 needed liver transplantation, in recent years, some of them have been successfully treated without it, also in the presence of encephalopathy [52,91].

Since ALF is a rare occurrence in a rare disease such as WD, available data on the management of ALF-WD patients are few and heterogeneous in relation to the severity of the disease, the combinations of treatments used (chelators, zinc, plasmapheresis, other supportive treatments), and the dosage of drugs.

In clinical practice, when the diagnosis of ALF-WD is made, careful staging of the patient is required with the help of available prognostic scores. Symptomatic supportive therapies should be promptly initiated and specific therapies with chelators and zinc salts should be considered. It is the opinion of some experts that chelators should be used at low initial doses and gradually increased. Based on the reported experiences, in patients with ALF-WD, a combination therapy including chelators and zinc salts could be used, spacing the administrations from each other to avoid interference between the drugs. Over the course of the days, it is mandatory to carefully monitor liver function and identify the appearance of signs of encephalopathy early so that the patient can be promptly included in the transplant list.

Below, we report the information available in the literature in order to detail the current knowledge about the management of ALF-WD.

Santos Silva et al. [105] reported a series of five pediatric patients with ALF-WD. One patient received D-penicillamine (25 mg/kg per day) as the first treatment; three were treated with D-penicillamine and zinc sulfate (10 mg/kg per day); and the fifth patient with trientine, 30 mg/kg per day. Three patients did not tolerate D-penicillamine and switched to trientine. One patient also needed plasmapheresis. Although four patients with a Nazer score ≥ 6 were candidates for liver transplantation, all were successfully treated with pharmacologic therapy alone. In another study by Eisenbach et al. [58], four of seven patients with ALF-WD were treated with chelating agents (three patients with D-penicillamine and one with trientine) and survived without liver transplantation. Other clinical and laboratory features are reported in Table 4.

Fang et al. [114] presented 41 patients with ALF-WD, of which 35 (3 with a mild grade of encephalopathy), treated with D-penicillamine and zinc, survived with native liver. In this study, zinc therapy was an adjunctive treatment, except in a patient treated with zinc alone, after plasmapheresis, due to a D-penicillamine allergy. Overall, the mortality rate due to ALF-WD was 7.3% (three patients died) and 7.3% (three patients) underwent liver transplantation [115].

In a meta-analysis by Wiggelinkhuizen M et al. [102], 10/13 patients (77%) with ALF-WD without encephalopathy were treated with D-penicillamine: two of them died and one underwent liver transplantation. The efficacy rate of medical treatment was comparable with that reported in a prospective study of a cohort treated at King’s College Hospital of London with either zinc or chelators or both. Only 4/14 patients needed liver transplantation, while the others were managed with medical therapy despite 1 patient presenting an NWI of 11 [15].

The use of zinc monotherapy in patients with acute liver disease has not been well documented, but nevertheless, some cases of successful treatment are available [115,116]. In particular, Haftu et al. described a case of an adolescent male with severe hepatic presentation of WD. Laboratory tests showed hyperbilirubinemia, coagulopathy, hypoalbuminemia, and deranged liver enzymes. The patient had a Keyser–Fleischer ring, confirmed by slit-lamp examination, low serum ceruloplasmin, and high 24 h urine copper (150 µg). His decompensated liver disease was successfully treated with zinc monotherapy and a low-copper diet. In this case, the choice of zinc monotherapy was due to the unavailability of chelators. After months of treatment with zinc, the patient experienced a normalization of the synthetic function of the liver with clinical improvement [117].

Steroid therapy failed to show a beneficial effect in ALF-WD [118], and other studies on their effectiveness are lacking.

### 6.1. Supportive Strategies

Plasmapheresis has been shown to rapidly reduce serum copper levels and in most cases was used as a bridge to liver transplantation so that it can be a therapeutic option for children and young adults presenting with ALF-WD [119,120,121,122]. The potential role of plasmapheresis was first recognized in 1914 by Abel et al. in children with ALF-WD [123]. Plasmapheresis, using fresh frozen plasma as a replacement fluid, can rapidly remove not only significant amounts of copper but also aromatic amino acids, ammonia, endotoxins, and other factors responsible for hepatic encephalopathy. Kido et al. described five patients with severe ALF-WD of which three patients presented encephalopathy grade I and recovered from ALF without liver transplantation [112]. Laboratory and therapeutic details are reported in Table 4. The authors concluded that in the group of ALF-WD patients surviving with native livers, favorable outcome was more likely associated with supportive treatments such as plasmapheresis rather than WD-specific therapy [112]. Vandriel SM et al. studied a large cohort of children and adolescents with ALF-WD. Notably, 11% of the subjects achieved recovery, while all other subjects underwent liver transplantation or died. In this cohort, plasmapheresis combined with D-penicillamine was the most frequent therapeutic strategy associated with recovery [59].

Some case reports demonstrated transplant-free survival after plasma exchange and subsequent chelation therapy, despite an NWI ≥ 11 [94]. Other therapeutic strategies included dialysis, albumin dialysis, and recirculating molecular adsorbent system (MARS) [124,125]. Nagata et al. compared the efficiency of removing copper with the different available methods applied simultaneously and showed higher efficiency of plasmapheresis versus continuous hemodiafiltration (20,600 μg of copper versus 3400 μg) [126].

MARS is a hemodialysis system coupled to a closed-circuit containing albumin-rich dialysate with a carbon filter and an anion exchanger. This system aims to mimic the detoxification mechanisms that occur at the hepatocyte membrane level and is also able to remove protein-bound toxins such as copper. MARS has been successfully used in patients with poor prognosis due to acute or chronic hepatic failure or type I hepatorenal syndrome [123,127]. Based on some cases [128,129,130] and WD clinical practice guidelines [23], MARS dialysis can stabilize the patient’s condition and can act as a bridge to liver transplantation. Reducing the copper load by MARS can confer a benefit in the management of acute decompensated WD [131,132], and sometimes several sessions of MARS may be necessary to achieve the therapeutic goal (cases in the literature report up to 18 sessions of MARS) [133].

Encephalopathy represents a negative prognostic factor in patients with ALF, linked to the accumulation of neurotoxic or neuroactive substances in the brain. Albumin dialysis is able to prevent these accumulations in patients with ALF-WD [134], causing improvement of the encephalopathy.

### 6.2. Liver Transplantation

Liver transplantation is considered the treatment of choice in patients with acute liver failure or decompensated chronic liver disease who do not have a response to medical therapy [135]. Patients without encephalopathy are more likely to avoid liver transplantation and be successfully treated with a combined strategy including chelators, zinc, and supportive therapies. Indications for liver transplantation must be individualized. Liver transplantation represents an optimal treatment for patients with ALF-WD, determining excellent long-term survival and a good quality of life [136,137,138,139]. Arnon R. et al. reported a large cohort of 90,867 patients transplanted between 1987 and 2008, of which 170 children and 400 adults had WD [140]. One- and five-year patient survival in WD children was 90.1% and 89%, respectively, compared to 88.3% and 86% in WD adults. These rates were higher if compared to those observed in children and adults transplanted for other causes in the same period [140]. Schilsky et al. analyzed 55 transplants performed in 21 patients with ALF-WD: the median survival time after liver transplantation was 2.5 years, the longest survival time was 20 years, and the one-year survival frequency was 79% [141].

As for the indications of liver transplantation, based on the guidelines and papers examined in this narrative review, WD patients with an NWI ≥ 11 have a high probability of needing a liver transplant and therefore should be referred to a transplant center or tertiary care unit and carefully monitored with regard to clinical, neurological, and laboratory aspects. Intensive treatment including WD-specific medications and supportive therapies should be promptly initiated. Based on the patient’s response, the clinical course over the days, and the availability of compatible donors, a decision will be made whether or not to transplant the patient. The concomitant presence of factors such as encephalopathy, coagulopathy, renal failure, hypoglycemia, or metabolic acidosis will favor the choice of liver transplant. In patients with an NWI score < 11 who have a higher probability of survival with native liver, close monitoring is advisable, and listing for liver transplantation is recommended in case of clinical worsening during observation. Maximum effort must be made to ensure the patient’s survival with a native liver. However, further studies are desirable for a more accurate selection of patients for liver transplantation and, in this context, the creation of biomarkers predictive of clinical deterioration could be useful.

### 6.3. Emerging Strategies

Emerging therapeutic strategies for WD include new chelators and treatments aiming to correct abnormal ATP7B expression or function. The main purpose of gene therapy in WD is to restore ATP7B-mediated hepatobiliary copper excretion [142]. Preclinical studies, using viral vectors with modified ATP7B constructs in models of WD, have shown restoration of copper balance and prevention of copper-induced liver injury [142]. These emerging treatments may be useful in combination with existing pharmacological treatments for WD. As for ALF-WD presentation, there are no new emerging therapeutic strategies to report.

## 7. Conclusions

**Figure 2:**
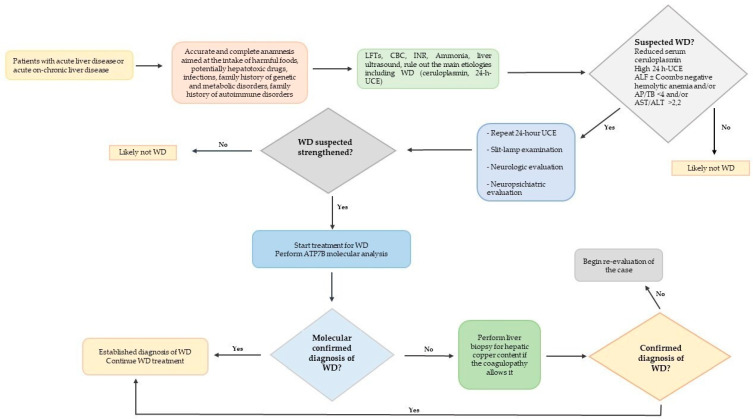
Proposed flow chart to approach pediatric patient with acute liver disease focused to diagnose Wilson’s disease. Abbreviations: LFTs, liver function tests; CBC, cell blood count; INR, international normalized ratio; WD, Wilson’s disease; UCE, urinary copper excretion; ALF, acute liver failure; AP, alkaline phosphatase; AST, aspartate aminotransferase; ALT, alanine aminotransferase [20].

In the context of the acute hepatic presentation of WD, ALF-WD represents a major clinical challenge. An orientation flowchart is proposed for the approach to pediatric patients with acute liver disease to facilitate the diagnosis of WD (Figure 2).

The diagnosis of WD in patients with acute liver disease requires the integration of multiple elements. It is limited by the fact that the only two highly specific diagnostic tests are time-consuming (molecular analysis) or rarely performed for severe coagulopathy (liver biopsy). However, in the near future, the evolution of genetic tests could ensure that the diagnosis of ALF-WD is facilitated by the possibility of rapidly knowing the presence or absence of mutations in the ATP7B gene. 

Once the diagnosis has been established, it is crucial to stage the liver disease severity and neurological involvement using the available scores. Patients with an NWI score ≥ 11 must be listed for liver transplantation, but in the meantime, supportive and specific therapy (chelators and zinc) must be started with close monitoring of the patient in order to verify if liver function gradually improves or whether there is a need for urgent liver transplantation [44]. In patients with an NWI score < 11, the chances that medical therapy will avoid liver transplantation are higher. As for the therapeutic management of patients with ALF-WD, there is a certain heterogeneity of information on how to assemble the supportive treatments (plasmapheresis, MARS, dialysis, albumin dialysis) and the specific drugs for WD (chelators, zinc) and which dosages to use. An aspect worthy of attention is represented by the initial dosage of chelators that generally should be started with a low dose to increase gradually, but in the WD-ALF setting, there are no clear indications. Even if zinc salts are classically indicated for asymptomatic or neurological WD patients, there are cases in which they were used as an ancillary therapy (in combination with chelators) in ALF-WD patients and anecdotal cases in which zinc monotherapy was administered. It seems reasonable to use zinc in combination with chelators, even if there are no univocal recommendations [97]. In the near future, in the management of patients with ALF-WD, more attention should be paid to the role of zinc therapy, as well as how to combine the different available treatments (specific and supportive) and the doses of each therapy.

Future research strategies are desirable to facilitate the diagnosis of WD and its management. Particularly, as a first research step, multicentric retrospective studies on cohorts of patients with ALF-WD could be useful to identify further elements suggesting the diagnosis, new prognostic markers, and the most effective therapeutic strategies among those tested.

## Figures and Tables

**Table 1:** Diagnostic score for Wilson’s disease in children based on reference values for age. Adapted from Ref. [48]. Abbreviations: KF, Kayser–Fleischer; AHA, autoimmune hemolytic anemia; ULN, upper limit of normal.

Points	Clinical Signs		Laboratory Signs			Histological Signs		Genetic Analysis
	*Neurological* *involvement*	*KF rings*	*Ceruloplasmin*	*Urinary copper*	*Coombs-negative AHA*	*Liver copper*	*Rhodanine stain*	*Mutation ATP7B*
−1						Normal (<50 μg/g dry weight)		
0	Absent	Absent	Normal (<20 mg/dL)	Normal (<40 μg/24 h)	Absent		Absent	No mutations needed
1	Mild		10–20 mg/dL	1–2 ULN (40–80 μg/24 h)	Present	<5 × ULN (50–250 μg/g dry weight)	Present	1 chromosome mutation
2	Severe	Present	<10 mg/dL	>2 × ULN (>80 μg/24 h) Normal but >5 × ULN (>200 μg/ 24 h after penicillamine challenge)		>5 × ULN (>250 μg/g dry weight)		
4								2 chromosomes mutations

**Table 2:** Prognostic scores for acute liver failure due to Wilson disease. Adapted from Ref. [14]. Abbreviations: Bil, bilirubin; AST, aspartate aminotransferase; INR, international normalized ratio; WBC, white blood cell count; Alb, albumin.

Nazer Score				New Wilson Index				
Bil, mg/dL (µmol/L)	INR	AST (IU/L)		Bil, mg/dL (µmol/L)	INR	AST (IU/L)	WBC (×10^9^/L)	Alb (mg/dL)
<5.8 (<100)	<1.3	<100	**0**	0–5.8 (0–100)	0–1.29	0–100	0–6.7	>4.5
5.9–8.7 (100–150)	1.3–1.6	100–150	**1**	5.9–8.7 (101–150)	1.3–1.6	101–150	6.8–8.3	3.4–4.4
8.8–11.7 (151–200)	1.6–1.9	151–200	**2**	8.8–11.7 (151–200)	1.7–1.9	151–200	8.4–10.3	2.5–3.3
11.8–17.5 (201–300)	1.9–2.4	201–300	**3**	11.8–17.5 (201–300)	2.0–2.4	201–300	10.4–15.3	2.1–2.4
>17.5 (>300)	>2.4	>300	**4**	>17.5 (>300)	≥2.5	>300	>15.4	≤2

**Table 3:** Current drug therapy for Wilson’s disease. Data from Refs [4,23,40,104].

	D-Penicillamine	Trientine	Zinc Salts
**Mode of action**	Increase in urinary copper excretion. Release of intracellular stores of metallothionein.	Increase in urinary copper excretion. Possible inhibition of dietary copper absorption.	Induction of copper-binding metallothioneins in gut and liver resulting in reduced intestinal absorption binding of copper to non-toxic compounds.
**Indications**	Initial treatment: symptomatic patients (initial neurological deterioration may occur). Maintenance treatment.	Initial treatment: symptomatic patients (initial neurological deterioration is less common). Maintenance treatment.	Initial treatment: asymptomatic patients, children with mild liver disease, neurological patients. Maintenance treatment.
**Dosage**	*Initial dose*: start with low dose and gradually increase up to 20 mg/kg/day in 2 or 3 doses. *Maintenance dose:* 10–15 mg/kg/day in 2 separate doses. To be modulated based on urinary copper excretion.	*Initial dose*: start with low dose and gradually increase up to 20 mg/kg/day in 2 or 3 doses. *Maintenance dose:* 10–15 mg/kg/day in 2 separate doses. To be modulated based on urinary copper excretion.	Age > 16 years and body weight > 50 kg: 50 mg three times a day. Age 6–16 years and body weight < 50 kg: 25 mg three times a day. Younger than 6 years of age: 25 mg twice a day.
**Administration Mode**	1 h before or 2–3 h after meals	1 h before or 2–3 h after meals.	1 h before or 2–3 h after meals.
**Parameters of treatment adequacy**	*Urinary copper:* 200–500 μg/24 h (3–8 μmol/24 h) on maintenance treatment.	*Urinary copper*: 200–500 μg/24 h (3–8 μmol/24 h) on maintenance treatment.	*Urinary copper*: <75 μg/24 h (<1.2 μmol/24 h) on maintenance treatment. *Urinary zinc*: >2000 μg/24 h (>30.6 μmol/24 h) on maintenance treatment. *Serum zinc:* >125 μg/dL (>1.9 μmol/dL) on maintenance treatment.
**Main side-effects**	Neurologic worsening (10–50%), hypersensitivity reactions, fever, neutropenia, thrombocytopenia, lymphadenopathy or proteinuria, lupus-like syndrome, skin lesions, antinuclear antibodies positivity.	Neurologic worsening (26%), anemia, skin alterations.	Nausea, gastritis, asymptomatic pancreatic hyperenzimemia.

**Table 4:** Overview of acute liver presentation in Wilson’s disease patients. Abbreviations: WD: Wilson’s disease; ALF: acute liver failure; F: female; M: male; TB: total bilirubin; ALT: alanine aminotransferase; AST: aspartate transaminase; AP: alkaline phosphatase; GGT: gamma-glutamyl transferase; INR: international normalized ratio; WBC: white blood cell; Hb: hemoglobin; HE: hepatic encephalopathy; KFR: Kayser–Fleischer ring; OLT: orthotopic liver transplantation; LT: liver transplantation; PE: plasmapheresis. N/A: not available.

Reference	Patients Number	Age at Presentation, Years	Clinical Manifestations	Laboratory Findings	Treatment and Outcome
Verma N. et al., 2014 [106]	1, F	5	Abdominal pain, jaundice, irritability, facial puffiness, drowsy, and asterixis	Hb 6 g/dL; direct Coombs test negative; TB/DB 53.4/34.1 mg/dL; serum ceruloplasmin 8.5 mg/dL; serum copper 340 μg/dL; 24 h urinary copper 440 μg	*Treatment:* plasma exchange, D-penicillamine, and zinc salts Family refused LT *Outcome:* died of acute pulmonary hemorrhage
Devarbhavi H. et al., 2014 [45]	61, 38 M with ALF on 145 WD’s children	9.7 ± 2.8	Ascites: 48(78.7%) Jaundice: 49 (80.3%) HE: 27 (44.3%) KFR: 53 (89.8%) Hepatomegaly: 38(62.3%) Splenomegaly: 35 (57.4%)	Hb 8.4 ± 2.1 g/dL; WBC 12578 ± 10585 × 10^3^/dL; platelets 1.15 ± 0.61 × 10^5^/dL; INR 4.3 ± 2.2; AST 279 ± 301 U/L; ALT 126 ± 206 U/L; TB 15.9 ± 13.6 mg/dL; DB 9.5 ± 9 mg/dL; ceruloplasmin 10.9 ± 8.6 mg/dL; serum copper 820 ± 798 μg/dL	*Treatment:* N/A *Outcome*: 33 (54.1%) patients died (22 with HE and 11 without HE)
Durand F et al.,2001 [107]	17, 5 M	16.6 (range 8–22)	Jaundice: 17 (100%) Ascites: 4 (23.5%) Fever: 8 (47%) HE: 2 (11.7%) Acute renal failure: 2 (11.7%) KFR: 13 (76.4%)	Ceruloplasmin: low in 12 patients and normal in the remaining; cupruria was above 5 µmol/24 h (equal to 317.7 µg/24 h) in 17 patients Family history of WD: 9 *Liver histology:* 13 cirrhosis; 3 chronic active hepatitis with mild fibrosis; 1 extensive fibrosis	*Treatment:* 11 treated with D-penicillamine 2 patients with HE were transplanted *Outcome:* 10/11 treated with D-penicillamine survived without LT and with subsequent normalization of liver function 4 died of HE
Eisenbach C et al., 2007 [58]	7, 0 M	20.1 ± 11.7	ALF: 7 (100%) KFR: 3 (42.8%) HE: 3 (42.8%) patients grade II; 4 (57.1%) grade I	ALT 53 ± 43 U/L; AST 87 ± 44 U/L; AST/ALT Ratio 2.3 ± 1.5; TB 23 ±19 mg/dL; INR 2.1 ± 0.4; serum AP 128 ± 89 U/L; AP/TB ratio 9.3 ± 8.9; albumin 29.8 ± 4.7 g/L; Hb 7.0 ± 2.2 g/L; ceruloplasmin 0.12 ± 0.08 g/L; urinary copper 93.4 ± 144 μmol/24 h; serum copper 28.1 ± 29.4 μmol/L 4 patients underwent genetic investigation with positive results	*Treatment:* 4 patients treated with chelating agents (3 D-penicillamine; 1 trientine) 3 patients underwent urgent OLT (2 of them presented HE grade II) *Outcome:* All patients were alive (3 after OLT)
Markiewicz-Kijewska M et al., 2008 [47]	13, 1 M	15.5 (range 6.4–21)	Weakness: N/A Abdominal pain: N/AJaundice: N/A HE: 11 (84.6%)	TB 42.24 mg/dL (range 4.5–71.6); INR 5.4 (range 2.9–10); AST 268.5 U/L (range 66–763); ALT 190.2 U/L (range 59–503)	*Treatment:* 1 patient: intensive chelating therapy and MARS 1 patient died without LTx due to brain damage 11 patients → LT *Outcome:* 10 patients were alive with good liver function (9 after LT and 1 without LT); 2 died in early periods after LT
Kido J. et al., 2017 [108]	4, 1 M	11 (range 6–15)	HE: 2 (50%) grade II and 2 (50%) grade I Fatigue: 3 (75%) Jaundice: 3 (75%) Abdominal distention: 2 (50%)	AST 106 U/L (range 72–151); ALT 34.75 U/L (range 17–64); TB 24.3 mg/dL (range 5.3–46.3); INR 2.32 (range 1.94–2.77); Hb 6.62 g/dL (range 5.2–7.6); ceruloplasmin 11.5 mg/dL (range 5.9–18.1); urinary copper 6982.25 μg/24 h (range 351–16592)	*Treatment:* 2 patients: D-penicillamine (1000 mg/die), plasma exchange, and for lack of improvement underwent LT 1 patient: continuous hemodiafiltration 1 patient: plasma exchange, continuous hemodiafiltration, and later development of cirrhosis and esophageal varix 1 patient: plasma exchange and treatment with zinc and trientine *Outcome:* N/A
Mainardi V. et al., 2019 [109]	6, 0 M	18 (range 12–22)	Jaundice: 6 (100%) HE: 6 (100%) grade I-II and in 3 (50%) progression to grade III-IV Acute Renal failure: 4 (66.6%) KFR: 2 (33.3%) hyperintense lesions at the basal ganglia: 1 (16.6%)	AST 156.83 U/L (range 83–250); ALT 51 U/L (range 15–119); TB 27.5 mg/dL (range 5.2–44); INR 4.2 (range 2–7); Hb 7 g/L (range: 5.1–9.0); ceruloplasmin 15.46 g/L (normal > 20 mg/dl ); urinary copper 1373.2 μg/24 h (normal < 100 μg/24 h)	*Treatment:* all 6 patients treated with D-penicillamine and listed for LT 3 patients underwent LT (2 patients survived and one died in the post-surgery period) *Outcome:* 2 patients died (1 for sepsis and the other for multi-organ dysfunction)
Garrido et al., 2022 [110]	4, 3 F	18 (range 13–22)	ALF: 4 (100%) KFR: 4 (100%) Hemolytic anemia: 2 (50%) Neurologic manifestations: 2 (50%)	Staging of liver fibrosis F4 (*Metavir score*): 4 patients *Liver biopsy:* cirrhosis and reticular collagen and confirming copper accumulation in 4 patients	*Treatment:* 4 LT *Outcome:* all patients are alive
Couchonnal E. et al., 2021 [111]	26, 18 F	12.2 ± 2.9	ALF: 26 (100%) KFR: 15 (58%)	AST 327.3 ± 299.4 IU/L; ALT 109.5 ± 102.7 IU/L; serum ceruloplasmin 11.6 ± 7.4 mg/dL; urinary copper excretion 57.4 µmol/24 h (2.0–254) equal to 3630.5 µg/24 h	*Treatment:* 26 LT *Outcome:* 2 patients died of immediate complications relative to LT
Kido J et al., 2018 [112]	5, 2 M	11 (range 6–15)	ALF: 5 (100%) HE: 3 (60%) patients Grade I and 2 (40%) patients Grade II Coombs-negative hemolytic anemia: 4 (80%) KFR: 3 (60%) Jaundice and general malaise: 3 (60%)	*Case 1:* Hb 9.5 g/dL; AST 146 U/L; ALT 67 U/L; INR 3.11; ceruloplasmin 7.0 mg/dL; urinary copper excretion 720 µg/24 h *Case 2:* Hb 7.6 g/dL; AST 72 U/L; ALT 17 U/L; INR 2.37; ceruloplasmin 18.1 mg/dL; urinary copper excretion 2731 µg/24 h *Case 3:* Hb 5.2 g/dL; AST 151 U/L; ALT 33 U/L; INR 2.23; ceruloplasmin 16.0 mg/dL; urinary copper excretion 16592 µg/24 h *Case 4:* Hb 9.8 g/dL; AST 261 U/L; ALT 57 U/L; INR 2.77; ceruloplasmin 6.0 mg/dL; urinary copper excretion 351 µg/24 h *Case 5*: Hb 6.8 g/dL; AST 120 U/L; ALT 25 U/L; INR 1.94; ceruloplasmin 5.9 mg/dL; urinary copper excretion N/A	*Treatment:* 3 patients survived without LT; 2 patients underwent LT (1 for bleeding from the esophageal varix) *Case 1:* zinc (3 mg/kg/die) and fresh frozen plasma; *Case 2:* PE, zinc, and trientine; *Case 3:* D-penicillamine (1000 mg/die) but for deterioration of consciousness, received PE without LT; *Case 4:* D-penicillamine (20 mg/kg/die) and after underwent LT; *Case 5:* D-penicillamine, zinc, and PE but for mental deterioration she underwent LT *Outcome:* N/A
Zou J et al., 2021 [113]	1, F	19	Jaundice; hepatosplenomegaly, paroxysmal colic in the upper right abdomen; skin damage; photosensitivity; stomachache, and acute neurovisceral symptoms ALF KFR Coombs-negative hemolytic anemia	AST 61.7 IU/L; ALT 42.5 IU/L; GGT IU/L 157.6; TB 464.0 μmol/L; Hb 89 g/L; INR 1.91; ceruloplasmin 66.1 mg/L; 24 h urine copper 3804 μg/24 h; Coombs test negative Mutation in intron 1 of *ATP7B* (c.51 + 2T > G) was found	*Treatment:* D-penicillamine (increased to 900 mg/die), zinc (150 mg/die), and PE *Outcome:* after one year, he developed worsening liver function subsequent to arbitrary discontinuation of drug therapy and he underwent LT
